# The Characteristics and Outcomes of Contralateral Non-Concurrent Hip Fractures: A Retrospective Study in Geriatric Patients

**DOI:** 10.3390/medicina60060928

**Published:** 2024-06-01

**Authors:** Sönmez Sağlam, Mehmet Arıcan, Zekeriya Okan Karaduman, Mücahid Osman Yücel, Erdem Değirmenci, Veysel Uludağ

**Affiliations:** Department of Orthopaedics and Traumatology, Faculty of Medicine, Duzce University, 81620 Duzce, Turkey; mehmetarican@duzce.edu.tr (M.A.); karadumano@hotmail.com (Z.O.K.); mucahidosmanyucel@gmail.com (M.O.Y.); erdem.degirmenci@kolanhastanesi.com.tr (E.D.); vuludag1365@outlook.com (V.U.)

**Keywords:** proximal femur fracture, opposite hip fracture, risk factor, inter-fracture interval

## Abstract

*Background and Objectives*: This study aimed to determine the relationship between non-simultaneous contralateral hip fractures, urban and rural differences, fracture localization, time between fractures, physiotherapy applications, comorbidity, and the second fracture outcomes. *Materials and Methods*: We retrospectively analyzed 107 patients aged 65 and older with proximal femur fractures (PFFs) who underwent surgery at Düzce University Medical Faculty between January 2010 and December 2022. High-energy fractures, pathological fractures, and patients with a history of old fractures were excluded. *Results*: The study included 66 females (61.7%) and 41 males (38.3%), with a mean age of 83.76 years. The mean interval between two fractures was 28.3 months. There was no statistical difference between the localization of the first and second fractures (*p* = 0.107). However, there was a significant difference in the first PFF localizations of patients living in rural areas (*p* = 0.023). Patients with heart failure, respiratory failure, osteoporosis, and cognitive impairment had shorter intervals between fractures (*p* < 0.001). *Conclusions*: This study shows that age, female gender, place of residence, comorbid diseases, and whether physical therapy is received after the first fracture are significant risk factors for a second hip fracture in patients over 65 years of age.

## 1. Introduction

Increasing life expectancy worldwide has led to a rise in the number of osteoporotic patients, making osteoporosis-related proximal femur fractures a leading cause of morbidity among the elderly. Osteoporosis weakens bones, making them more susceptible to fractures even from low-energy traumas such as falls. In 2010, the global number of hip fractures was estimated to be approximately 2.7 million cases per year. Conservative estimates predict that this number will increase to 4.5 million by 2050 [1]. This substantial rise poses a significant public health challenge, particularly as hip fractures are associated with considerable morbidity and mortality.

A large proportion of patients with femur fractures lose a significant portion of their independence in daily activities, suffer from prolonged hospitalization, require long-term care in treatment centers, need walking aids, or become immobile [2,3,4]. These consequences not only affect the patients’ quality of life but also place a considerable burden on healthcare systems. Patients who suffer a low-energy hip fracture are at a high risk of sustaining a contralateral hip fracture in the subsequent period [5,6]. The incidence of non-concurrent bilateral hip fractures reported in the literature varies widely, ranging from 1.7% to 15% [7,8]. It is estimated that 2% to 11% of individuals with an initial hip fracture will experience a second hip fracture within 1 to 7 years [9].

The scientific community has shown increasing interest in innovative techniques to reduce surgical time, minimize errors, and lower complications associated with hip fractures [10]. However, there is still limited knowledge about the impact of the location of primary fractures and the surgical techniques used on the risk factors for second hip fractures. Mortality rates remain a significant concern, with reported rates of 10% in the first month and up to 35% at the end of the first year post-surgery [11]. Different studies have reported one- and five-year mortality rates of 15.9% and 45.4%, respectively, for patients after their first hip fracture. For patients with a previous contralateral hip fracture, mortality rates one and five years after the second event were even higher, at 24.1% and 66.5%, respectively [12]. Another study concluded that the cumulative incidence of mortality was approximately 9% after one year and 20% after five years [13,14].

Given the significant implications of second hip fractures, it is crucial to identify and understand the risk factors that contribute to their occurrence. This knowledge is essential for developing effective prevention strategies. The aim of this study was to retrospectively evaluate patients who presented to our hospital with contralateral hip fractures at different times. By analyzing the risk factors leading to a second hip fracture after the initial fracture, we aim to provide insights that could help in formulating strategies to prevent secondary fractures, ultimately improving patient outcomes and reducing the burden on healthcare systems.

## 2. Materials and Methods

In our study, 1697 patients over 65 years of age who were admitted to the Orthopedics and Traumatology Clinic of Düzce University Medical Faculty Hospital between January 2010 and December 2022, diagnosed with proximal femur fractures (intracapsular, extracapsular, and subtrochanteric) and treated surgically, were retrospectively analyzed using archive records. We evaluated five diseases—osteoporosis, heart failure, diabetes, cognitive impairment, and respiratory diseases—as major comorbidities. The presence of each disease was added to the list of comorbidities, with a score of 1 point given for each disease, resulting in a total score between 0 and 5. Necessary permissions were obtained from the Düzce University Ethics Committee (No: 2024/55).

We identified 123 patients who had previously undergone osteosynthesis or arthroplasty for another hip fracture. Of these, 16 patients were excluded: 8 patients with old fractures, 4 patients with pathological fractures, and 4 patients with hip fractures due to multiple trauma. A total of 107 patients were included in our study.

The fractures were classified according to the AO Foundation/Orthopaedic Trauma Association classification system [15]. Inclusion criteria were patients aged 65 years and older with a proximal femur fracture who underwent surgery (osteosynthesis or arthroplasty) for a contralateral hip fracture. Exclusion criteria included high-energy fractures, pathological fractures, patients with a history of old fractures, and patients with hip fractures due to multiple trauma.

The relationship between the first hip fracture and subsequent contralateral hip fracture, age, gender, place of residence, time elapsed between the two fractures, first fracture localization, second fracture localization, and preferred surgical method were examined. Additionally, the effect of the number of comorbid diseases on the duration between fractures, the effect of physical therapy after the first fracture on fracture duration, the effect of preoperative waiting time on ICU length of stay, and the relationship between ICU length of stay and early mortality were analyzed.

Our hypothesis was that patients with more comorbidities and those who did not receive physical therapy after the first fracture would have shorter intervals between fractures and higher ICU lengths of stays, contributing to higher early mortality rates. The primary endpoint was the interval between the first and second hip fractures. Secondary endpoints included the impact of comorbidities and physical therapy on the interval between fractures, ICU length of stay, and early mortality rates.

### Statistical Analysis

Mean Standard Deviation and Median (Min–Max) were given in descriptive statistics for continuous data, and number and percentage values were given in discrete data. The Shapiro–Wilk test was used to examine the conformity of continuous data to normal distribution. One-way analysis of variance (ANOVA) was used for comparisons of patient age between fracture localization groups, and Kruskal–Wallis analysis of variance was used for comparisons of time between two operations between fracture localization groups. The Chi-Square test was used for group comparisons of nominal variables (cross tabulations). The McNemar test was used to examine the differences between the first and second operations of nominal variables. Correlations between preoperative waiting times, the number of comorbid diseases, and intensive care unit (ICU) hospitalization times were analyzed using Spearman’s correlation coefficient. The IBM SPSS version 20 (Chicago, IL, USA) program was used in the evaluations, and *p* < 0.05 was accepted as the limit of statistical significance.

## 3. Results

A total of 1697 patients with proximal femur fractures between 1 January 2010 and 31 December 2022 were included in the study. A total of 107 patients with proximal femur fractures at different times who met the inclusion and exclusion criteria were included in the study. Of these, 32 (29.9%) patients had intracapsular fractures, 68 (63.6%) patients had extracapsular fractures, and 7 (6.5%) patients had subtrochanteric fractures. Of the patients, 66 (61.7%) were female, 41 (38.3%) were male, and 70.1% lived in the city center. The mean age at the second fracture was 83.76 ± 6.07 (66–96) years, and the mean interval between fractures was 28.39 ± 12.15 (1–75) months (Table 1). In our study, 10 (9.3%) of the second hip fractures occurred within the first year following the first hip fracture, and 85 (79.4%) patients developed a second fracture within the first 36 months (Table 1).

There was no statistical difference between the first and second fracture localizations (*p* > 0.05) (Table 2). The second fracture localization was extracapsular in 43 of 54 patients with an extracapsular first fracture, intracapsular in 20 of 46 patients with an intracapsular first fracture, and subtrochanteric in 2 of 7 patients with a subtrochanteric first fracture. As a result, the first and second fracture localizations were the same in 69 of 107 patients. There was no difference in the time elapsed between the two fractures between patients with extracapsular, intracapsular, and subtrochanteric first hip fracture localizations (*p* > 0.05).

Among patients with extracapsular, intracapsular, and subtrochanteric first hip fracture localizations, there was no statistically significant difference between men and women in terms of age and between first and second hip fracture localizations (*p* > 0.05). There was a difference between the first hip fracture localizations of patients living in urban and rural areas (*p* < 0.05). In rural patients, the rate of intracapsular hip fracture localization was higher and the rate of extracapsular localization was lower. There was no difference between the second hip fracture localizations of patients living in urban and rural areas (*p* > 0.05). Among the patients with a second hip fracture, there were 35 patients who had no physical therapy outpatient visits after the first fracture, 44 patients who had physical therapy outpatient visits, and 28 patients who received inpatient physical therapy. There was a statistically significant difference in the time elapsed between the two fractures in these patients (*p* < 0.001). Patients who did not apply for physical therapy after the first fracture had a shorter interval between the two fractures than those who applied to physical therapy outpatient clinic or received inpatient physical therapy.

There was no difference in the time interval between two fractures between patients who had physical therapy as outpatients or inpatients (*p* = 0.056). Patients with physical therapy outpatient visits and inpatients receiving inpatient physical therapy were more likely to have an interval between two fractures of 25–36 months and more than 36 months compared to patients with no physical therapy visits (Table 3). The rates of osteoporosis (67.3%), diabetes mellitus (72%), heart failure (54.2%), respiratory system diseases (52.3%), and cognitive impairment (40.2%) were higher. The most common comorbidity was DM and the least common comorbidity was cognitive impairment. In 50 patients, the number of comorbid diseases was two or less, while in 57 patients, it was three or more. There was a difference between the time between two fractures in patients with two or less comorbid diseases and patients with three or more comorbid diseases (*p* < 0.001). Patients with three or more comorbid diseases had less time between two fractures. There was a difference between patients with two or less comorbid diseases and patients with three or more comorbid diseases in terms of the time between two fractures being 24 months or less, between 25 and 36 months, and more than 36 months (*p* < 0.001). Patients with three or more comorbid diseases had a higher rate of 24 months or less between two fractures (Table 4). The mean preoperative waiting time was 3.55 ± 1.39 days. Intensive care unit (ICU) hospitalization was detected in 85% of the patients. The mean ICU hospitalization time of patients with ICU hospitalization was 5.41 ± 2.48 days. It was found that 12 (11.2%) of the patients died in the early postoperative period (Table 5). There was a difference between the time between two fractures in patients without osteoporosis and patients with osteoporosis (*p* < 0.01). The time between two fractures was shorter in patients with osteoporosis. Patients with heart failure had shorter times between two fractures than patients without heart failure (*p* < 0.05). Patients with respiratory system disease had shorter intervals between two fractures than patients without respiratory system disease (*p* < 0.001). Patients with cognitive impairments had shorter intervals between two fractures than patients without cognitive impairments (*p* < 0.001).

The ICU length of stay was not different between patients with osteosynthesis and arthroplasty (*p* > 0.05). There was a positive correlation between preop waiting times and ICU length of stay (r = 0.763 *p* < 0.001). As the preop waiting times of the patients increased, the duration of ICU hospitalization also increased. There was a positive correlation between the number of comorbid diseases and ICU length of stay (r = 0.305 *p* < 0.01). As the number of comorbid diseases increased, the duration of ICU stay also increased. The preoperative waiting times of 12 patients who had in-hospital deaths before hospital discharge were longer than those who survived (*p* < 0.001).

## 4. Discussion

In this study, we determined that age, gender, place of living, chronic diseases, and physiotherapy may be risk factors for the development of a second hip fracture in geriatric patients. Recent years have seen an increase in the incidence of hip fractures and patient survival rates due to rising life expectancy [16]. However, as existing risk factors continue to persist after treatment, it is anticipated that the incidence of consecutive hip fractures will also rise. The incidence of non-concurrent contralateral hip fracture in our study was 10.9%, which supports the 2–20% incidence reported in the literature [5,17].

Many studies have reported that the risk of a contralateral hip fracture is highest within the first 12 months following the initial fracture, after which, the risk decreases [14,18]. In another study, Sawalha et al. [12] reported that 70% of consecutive hip fractures occurred within 3 years after the first fracture. In our study, the second hip fracture occurred in three patients within 3 months (27–82 days) after the first fracture, a period during which bone healing was not complete. This may be attributed to inadequate gait rehabilitation and a new minor trauma leading to a second hip fracture. Furthermore, 9.3% of the fractures occurred within the first year following the initial hip fracture, and 84 patients (79%) experienced a second hip fracture within the first 3 years. The interval between the two consecutive hip fractures in our patients was 28.3 months, consistent with the literature. Fracture localization caused no statistical difference in the time between fractures.

With an aging population, hospital admissions for both first and second hip fractures are likely to increase significantly. Francesco Bosco et al. [19] reported that male gender was statistically more common in patients with a contralateral hip fracture within three years, and the duration of re-fracture was shorter in these patients. However, other studies have challenged these findings. For example, Patrick Nolan et al. [20] found that patients presenting with a second hip fracture were mostly female. Gaumetou et al. [21] also showed that first and second hip fractures were predominantly in women. Similarly, our study found that women had a higher risk of a second hip fracture. Additionally, it has been emphasized that most patients admitted for a second hip fracture live in urban centers, highlighting the role of urban living in bone and joint health protection.

Hip fractures are highly associated with osteoporosis and are triggered by simple traumas such as falls. Therefore, risk factors for falls overlap significantly with those for hip fractures. Liu et al. [22] published a meta-analysis evaluating risk factors for a second hip fracture in the elderly population and concluded that nursing home residency, female gender, cognitive impairment, osteoporosis, visual impairment, dizziness, and cardiac and respiratory diseases were all associated with an increased risk of a second hip fracture. Our study is consistent with these risk factors. The American Anesthesia Society (ASA) score, used as an indicator of comorbidity, poor prognosis, and higher mortality risk, also plays a crucial role [23]. In this study, 95 patients (88%) with a high ASA score of 3 or 4 were identified, and 91 patients (85%) required postoperative intensive-care follow-up. The high ASA scores and the need for intensive care can be linked to the fact that our hospital is the only one with a tertiary intensive care unit and a cardiology intensive care unit in the region, resulting in a significant number of emergency referrals.

The most common comorbidity in our study was diabetes mellitus, while cognitive impairment was the least common. Of the 107 patients with a second hip fracture, 50 had two or fewer comorbidities, and 57 had three or more. Patients with two or fewer comorbidities had a significantly longer time to a second fracture than those with three or more (*p* < 0.001). These results suggest that comorbidities negatively impact both the first and second fractures and the interval between them. Unlike other comorbidities, diabetes did not affect the time between the two fractures (*p* > 0.05), potentially due to other comorbidities having a more pronounced effect on the brain and musculoskeletal systems.

Of the contralateral second hip fractures, 64.4% were of the same type as the initial hip fracture (40.18% extracapsular, 22.42% intracapsular, and 1.86% subtrochanteric). This rate aligns with the existing literature, which reports figures ranging from 64% to 83%. Shabat et al. [7] suggested that each patient’s unique bone architecture and gait pattern result in similar types of falls and fracture localizations. The similar fracture patterns observed in different hips at different times indicate that unchangeable risk factors and comorbidities persist similarly after the first fracture until the second. Future prospective multicenter studies with more cases, including bone density measurements after the first fracture, may be more useful in determining the potential locations of second fractures. The similarity in surgical methods chosen for the first and second fractures also reflects the existing literature [24]. Our study found no significant difference between the types of surgery for the first and second fractures, supporting the literature.

Jeffrey Chirk Fan Lau et al. [23] reported that patient compliance with bisphosphonate therapy following the first fracture increased from 13.5% in the first fracture group to 23.8% in the contralateral hip fracture group, resulting in a decrease in second hip fractures. Studies emphasizing the importance of achieving optimal bone health through bisphosphonates are common [24]. Our study found no difference between the first and second fracture localizations among urban residents, but those living in rural areas had higher rates of intracapsular first fracture localizations, with a statistically significant difference, though there was no difference in second fracture localizations. The difference in localization between the first and second fractures may be explained by supplementary medical treatments (vitamin D, bisphosphonate, calcium) used after the first fracture, although there are insufficient archival records.

Fukushima et al. [25] reported that patients with second hip fractures had worse subsequent mobility compared to those with unilateral hip fractures. S. Sawalha et al. [12] found that patients included in a planned rehabilitation program after their first fracture performed better after their second fracture. Our study retrospectively analyzed whether patients received inpatient or outpatient physical therapy after their first fracture. Patients without physical therapy were more likely to have an interval of 24 months or less between the two fractures compared to those who received physical therapy. There was no statistical difference between inpatient and outpatient physical therapy. These results suggest that rehabilitation or preventive medical treatment after the first fracture may be crucial in reducing the risk of a second fracture and prolonging the interval between fractures.

Many studies report the gender-specific and age-specific incidence of second hip fractures and the resulting mortality and time to a second fracture. Comorbidities such as dementia [9,26], Parkinson’s disease, audiovisual impairment, advanced age, body mass index, location, bone condition, Singh index, physical function, fracture location, and complications are frequently cited as risk factors [27,28]. Juhàsz et al. [29] showed that living in the capital city, hip arthroplasty after the first fracture, female gender, and advanced age were risk factors for contralateral hip fracture. In another study, Zhu et al. [30] reported in a meta-analysis that patients who were female, living in a nursing home, had dementia, visual impairment, Parkinson’s disease, cardiovascular, and respiratory diseases had a higher risk of contralateral hip fracture.

Data in the literature show 1- and 5-year mortality rates ranging between 15.9% and 45.4% for the first hip fracture, while mortality rates 1 and 5 years after the second event in patients with a previous contralateral hip fracture are 24.1% and 66.5%, respectively [29]. Another cumulative study concluded that this incidence was approximately 9% at one year and 20% after five years [13]. In our study, premature death occurred in 12 patients (11.2%) during the follow-up period of postoperative hospital intensive care and inpatient hospitalization. The most important and statistically significant reason for the high mortality rate, which was similar to the literature, was the long preoperative waiting time for intensive care after the second fracture in patients who died (*p* < 0.001).

The important limitations of our study, which covers approximately 12 years, include the retrospective review of data from a single center, inadequate archival documentation due to different hospitals treating the first fracture, and the inability to analyze bone-preservation methods such as bisphosphonates, calcium, and vitamin D supplements that may have been used after the first fracture.

## 5. Conclusions

In conclusion, this study reveals that various risk factors play an important role in the interval between the first and second hip fractures in geriatric patients. The risk of a second hip fracture was higher in patients with advanced age, female gender, multiple comorbidities, living in urban centers, and those who did not receive appropriate physical therapy after the first fracture. Identifying these high-risk groups is crucial for developing effective hip-fracture-prevention strategies. Addressing these factors through targeted interventions could potentially reduce the incidence and improve outcomes of second hip fractures in the elderly population.

## Figures and Tables

**Table 1:** Demographic and clinical characteristics of the patients.

	Mean ± SD Median (Min–Max)
Age (years) mean ± SD, (min, max)	83.76 ± 6.07 84 (66–96)
Time between two fractures (months)	28.39 ± 12.15 26 (1–75)
Number of comorbid diseases	2.85 ± 1.24 3 (1–5)
	n (%)
Gender	
Male	66 (61.7)
Female	41 (38.3)
Place of living	
City center	75 (70.1)
Rural areas	32 (29.9)
Physical therapy	
None	35 (32.7)
Polyclinic examination	44 (41.1)
Inpatient physiotherapy	28 (26.2)
Second fracture time	
<24 months	41 (38.3)
25–36 months	44 (41.1)
>36 months	22 (20.6)
Number of comorbid diseases	
≤2	50 (46.7)
≥3	57 (53.3)
1. Operation fracture localization	
Extracapsular	54 (50.5)
Intracapsular	46 (43)
Subtrochanteric	7 (6.5)
1. Operation type	
Osteosynthesis	61 (57)
Arthroplasty	46 (43)
2. Operation fracture localization	
Extracapsular	68 (63.6)
Intracapsular	32 (29.9)
Subtrochanteric	7 (6.5)
2. Operation type	
Osteosynthesis	74 (69.2)
Arthroplasty	33 (30.8)

**Table 2:** Comparison of fracture localizations of patients living in the center and rural areas.

	City Center	Rural Areas	
	n (%)	n (%)	*p*
Hip fracture localization 1			
Extracapsular	44 (58.7)	10 (31.2)	0.023 *
Intracapsular	27 (36)	19 (69.4)	
Subtrochanteric	4 (5.3)	3 (9.4	
Hip fracture localization 2			
Extracapsular	52 (69.6)	16 (50)	0.107
Intracapsular	18 (24)	14 (43.8)	
Subtrochanteric	5 (6.7)	2 (6.2)	

* Chi-Square/Fisher’s Exact test.

**Table 3:** Comparison of time interval between two fractures with comorbidities.

	Time between Two Fractures		
	Mean ± SD	Median (Min–Max)	*p*
Osteoporosis			
None	33.94 ± 10.36	34 (20–56)	**0.001** ^a^
Yes	25.69 ± 12.11	25 (1–75)	
Diabetes mellitus (DM)			
None	31.83 ± 12.27	31 (8–62)	0.067 ^a^
Yes	27.05 ± 11.92	25 (6–75)	
Heart failure			
None	31.51 ± 11.64	31 (8–62)	**0.014** ^a^
Yes	25.76 ± 12.05	24.5 (6–75)	
Respiratory diseases			
None	33.76 ± 11.53	35 (8–675)	**<0.001** ^a^
Yes	23.50 ± 10.62	22.5 (6–69)	
Cognitive impairment			
None	32.44 ± 11.03	33 (12–62)	**<0.001** ^a^
Yes	22.37 ± 11.33	21 (1–75)	

^a^: independent sample *t* test.

**Table 4:** Comparison of patient characteristics and the proportion of time between two fractures of 24 months, 24–36 months, and more than 36 months.

	Time between Two Fractures			
	≤24 Months	25–36 Months	>36 Months	
	n (%)	n (%)	n (%)	*p*
Place of living				
City center	27 (40.9)	28 (42.4)	11 (16.7)	0.440 ^c^
Rural areas	14 (34.1)	16 (39.0)	11 (26.8)	
1. Operation type				
Osteosynthesis	20 (32.8)	28 (45.9)	13 (21.3)	0.375 ^c^
Arthroplasty	21 (45.7)	16 (34.8)	9 (19.6)	
Physical therapy				
None	26 (74.3)	8 (22.9)	1 (2.9)	**<0.001** ^c^
Polyclinic examination	10 (22.7)	24 (54.5)	10 (22.7)	
Inpatient physiotherapy	5 (17.9)	12 (42.9)	11 (39.3)	
Number of comorbid diseases				
≤2	7 (14.0)	26 (52.0)	17 (34.0)	**<0.001** ^c^
≥3	34 (59.6)	18 (31.6)	5 (8.8)	

^c^: Chi-Square tests.

**Table 5:** Comparison of age and characteristics of living patients and patients with Ex.

	Living		Ex		
	Mean ± SD Median (Min–Max)		Mean ± SD Median (Min–Max)		*p*
Age	83.87 ± 6.15 84 (66–96)		82.83 ± 5.57 81.5 (73–93)		0.420 ^d^
Preop waiting time	3.37 ± 1.34 3 (1–7)		5.00 ± 0.85 5 (4–7)		**<0.001** ^d^
	**n**	**%**	**n**	**%**	
Number of comorbid diseases					
≤2	47	94.0	48	84.2	0.109 ^d^
≥3	3	6.0	9	15.8	
2. Operation type					
Osteosynthesis	65	87.8	30	90.9	0.751 ^d^
Arthroplasty	9	12.2	3	9.1	

^d^: Mann–Whitney U test.

## Data Availability

All data related to the study are stored electronically. The data can be obtained from the corresponding author upon request.
